# Preparticipation screening in young female elite ice hockey players

**DOI:** 10.3389/fcvm.2024.1461028

**Published:** 2024-12-23

**Authors:** Alexander Mohl, Janis Pongratz, Selina Muxel, Manuel Berger, Michael Berr, Bastian Schneider, Anna Schlichting-Knoob, Ulrich Platz, Uwe Dorwarth, Sebastian Rogowski, Ellen Hoffmann, Florian Straube

**Affiliations:** ^1^ Department of Cardiology and Internal Intensive Care Medicine, Heart Center Munich-Bogenhausen Munich Municipal Hospital Group, Munich, Germany; ^2^ Department of Cardiology, Cardio Centrum Düsseldorf, Düsseldorf, Germany; ^3^ Department of Cardiology, Cardiology Practice, Traunreut, Germany

**Keywords:** preparticipation screening, sudden cardiac death, prevention, athletes, ice hockey, females

## Abstract

**Objectives:**

The occurrence of sudden cardiac death (SCD) in competitive athletes has led to a discussion about appropriate preparticipation screening models. The role of an electrocardiogram (ECG) in routine testing remains controversial in current guidelines. Furthermore, data on cardiac findings and the prognostic utility of screening strategies in young female elite ice hockey is scarce.

**Methods:**

Female elite ice hockey players were enrolled in the open prospective “General Evaluation Program for Arrhythmia-Related Death in Athletes” (GEPARD) registry from 2008 to 2018. A staged preparticipation screening was performed. The main goal was to determine the prevalence of SCD conditions and identify effective screening tools. The secondary aim was to study baseline results and follow-ups on a unique subgroup of young female ice hockey players.

**Results:**

A total of 88 female ice hockey players, mean age 16 ± 1 years, were prospectively enrolled. The prevalence of conditions potentially leading to SCD during competition was 3.4% (3/88). The 12-lead ECG led to the diagnosis of one congenital long QT and one acute myocarditis and showed a number needed to screen of 44, with a specificity of 98%. One athlete demonstrated a relevant pericardial effusion on echocardiography, which was related to acute toxoplasmosis. No cases of SCD occurred during long-term follow-up.

**Conclusion:**

The subgroup of young female ice hockey players showed a notable prevalence of athletes “at risk” of 3.4%, which indicates the importance of preparticipation screening that features a 12-lead ECG as the most important component.

## Introduction

Sudden cardiac death (SCD) in competitive sports is a profoundly tragic event, typically affecting young, seemingly healthy athletes (1). Since 1999, when two professional male ice hockey players tragically died from SCD during a sport event, SCD has become a significant topic of concern in the professional ice hockey community (Table 1).

**Table 1:** Sudden cardiac death in professional ice hockey sport.

Name (year of age)	Gender	Year of death	Club/carrer	Situation/information on cause of death
Stéphane Morin (29)	M	1998	Berlin Capitals	Morin felt unwell during a game and collapsed on the bench. Autopsy: undiagnosed chronic bronchitis, previous unknown myocardial infarction and dilated heart
Chad Silver (29)	M	1998	Swiss National League, HC Fribourg-Gottéron and ZSC Lions Zurich	Cardiac death at home. Autopsy: Heart failure
Sergejs Žoltoks (32)	M	2004	Latvia, NHL Klub Riga 2000	HCM, collapsed while watching game at bench, died later because of heart failure
Darcy Robinson (25)	M	2007	Wilkes-Barre/Scranton Penguins Asiago HC, NHL draft Pittsburg Penguins	Heart attack during game: It was later discovered that Robinson had a rare heart condition that factored into his heart attack
Mickey Renaud (19)	M	2008	captain of the Windsor Spitfires, and a fifth round draft pick for the Calgary Flames	SCD due to HCM
Alexei Cherepanov (19)	M	2008	Omsk Avangard (KHL, Russia).	SCD, reports on chronic myocarditis, hypertophic cardiomyopathy, heart failure
Gábor Ocskay (33)	M	2009	Alba Volán Székesfehérvár in Hungary	In 2004, he was diagnosed with a heart disease and sidelined for four months, but later has received a medical permission to continue his career. He died of a heart attack
Igor Misko (23)	M	2010	Played for SKA St. Petersburg of the Kontinental Hockey League.	Cardiac arrest while driving, heart failure
Markus Wächter (19)	M	2010	Played for ESV Kaufbeuren (Germany)	Died in a hospital after collapsing following a bodycheck in a national junior league game. It was later announced that Wächter suffered from a heart condition
Jordan Boyd (16)	M	2013	Played for Acadie–Bathurst Titan in the Quebec Maritimes Junior Hockey League	Collapsed on the ice during training camp, and died as a result of an undiagnosed inherited heart disease ARVC
Nick Egan (21)	M	2014	Estevan Bruins of the Saskatchewan Junior Hockey League	Cardiac arrest
Ondřej Buchtela	M	2020	HC Benátky nad Jizerou. Played in the 2017 IIHF World U18 Championships for the Czech Republic	Heart cancer
Boris Sádecký (24)	M	2021	Bratislava Capitals	Myocarditis. Heartburn the day before. He collapsed during a game in Austria, and died the next morning

ESV, ice sport club; HC, hockey club; HCM, hypertrophic cardiomyopathy; IIHF, international ice hockey federation; KHL, continental hockey league; M, male; NHL, national hockey league; SCD, sudden cardiac death; SKA, sport club of the army; U18, under 18 years of age.

Chronologically arranged list of professional ice hockey players who died of sudden cardiac death between 1990 and 2024, along with the relevant information. The list was compiled using an internet search and an analysis of the wikipedia.org database, and makes no claim to completeness or accuracy. These cases highlight the importance of thorough medical examination and emergency procedures in ice hockey, to prevent similar tragic events in the future. The list does not include any female athletes who died of sudden cardiac death. The search was expressly carried out without regard to gender, and the reason for the exclusively male victims of these high-profile sudden death cases is unclear.

There is already substantial data on the increased risk, pathogenesis, and appropriate prevention of sudden cardiac death in competitive sports. However, most of these studies have predominantly focused on male athletes (1–3). Due to the increasing proportion of women in competitive sport, a gender-specific discussion is necessary. As existing studies showed women to have a 2–25 times lower risk of SCD than men (3–6), there may be less awareness of preparticipation screening for SCD in female sports.

Several studies indicate that the profile of women experiencing SCD differs from that of men in terms of underlying causes, clinical presentations, and outcomes. This necessitates further investigation to gather more data regarding the functionalities and performance of the female heart in competitive sports. Consequently, this will aid in developing appropriate preparticipation tests for all athletes (7, 8).

In this field there is an ongoing debate about the role of the 12-lead ECG as a fixed part of preparticipation screening between the American Heart Association (AHA), which is opposed due to the cost and the risk of false positive results (9–11), and the European Society of Cardiolgy (ESC), which clearly supports the use of the 12-lead ECG in screening after studies showed a significant reduction in SCD in athletes following its introduction as a mandatory screening test (1, 3, 12). This question might also need to be revisited with regard to gender differences.

The main objective of the study was to evaluate a preparticipation testing model to detect cardiovascular abnormalities that could potentially result in SCD and to define their prevalence in the special subgroup of female adolescent athletes. The secondary objective was to provide a unique baseline data-set of young female elite ice hockey players collected during preparticipation screening, and to provide follow-up data.

## Methods

### Study design

The “General Evaluation Program for Prevention of Arrhythmia Related Deaths in Athletes” (GEPARD) is an open, prospective, observational registry including competitive athletes who undergo preparticipation screening. The present study enrolled and analyzed all adolescent female ice hockey players referred from the German Female National Ice Hockey Team between 2008 and 2018. The study was approved by the ethical committee of the Bavarian Chamber of Physicians and follows the principles of the Declaration of Helsinki. All participants had to sign an informed consent form.

### Study protocol

**Figure 1:**
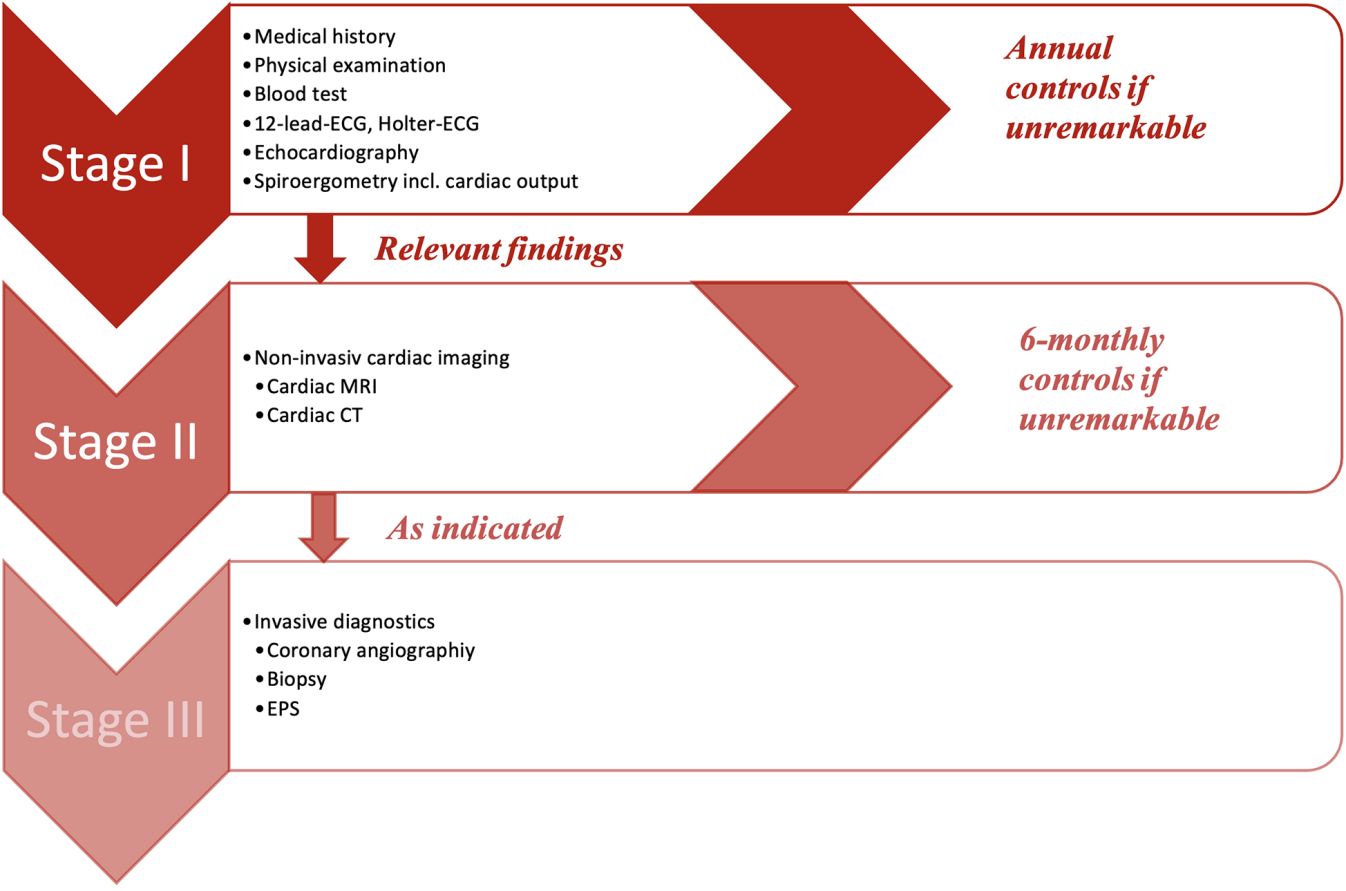
GEPARD multi-stage screening model. CT, computed tomography; ECG, electrocardiogram; EPS, electrophysiology study; MRI, magnetic resonance imaging.

The GEPARD protocol follows a preparticipation screening model, which consisted of three stages (Figure 1) and includes a similar diagnostic workup for assessing athletes with arrhythmias and/or suspected cardiomyopathies as previously described (13, 14).

Every player had to undergo stage I, which included the evaluation of the medical history, a physical examination, a blood test, a 12-lead-ECG, a ≥18 h of Holter-ECG and a transthoracic echocardiography. In addition to screening for SCD during stage I, a spiroergometry was also performed with an Innocor® inert gas rebreathing system by COSMED. This was done to provide additional practice control and to assess the level of physical performance, including maximum oxygen consumption (VO2max), aerobic and anaerobic thresholds, and cardiac output. A step-up protocol was applied with 3-min intervals starting at 50 W with 25 W steps of increase. Measurements of CO was performed at baseline, sub-maximum, and maximum workload. Lactate measurements were taken at the end of each step. ECG monitoring was performed continuously throughout the examination. The recordings were printed out at the end of each stage.

If no abnormalities were detected, the athlete was cleared for sport and competition. In case of any suspicious findings, stage II of the study protocol became applicable, which contained further non-invasive cardiac imaging like cardiac magnetic resonance imaging (MRI) or computed tomography (CT) as indicated. Stage III included all invasive further diagnostic approaches (i.e., cardiac catheterization, electrophysiological study, myocardial biopsy), and was performed as clinically indicated.

### Data analysis

Data was collected and analyzed on the secure institutional server using Microsoft Access, Microsoft Excel and IBM SPSS (USA). In accordance with the Shapiro-Wilk test, continuous variables are expressed as means with standard deviations (SD) or as medians with quartiles. Categorical data are shown as numbers and percentages.

## Results

### Participants

**Figure 2:**
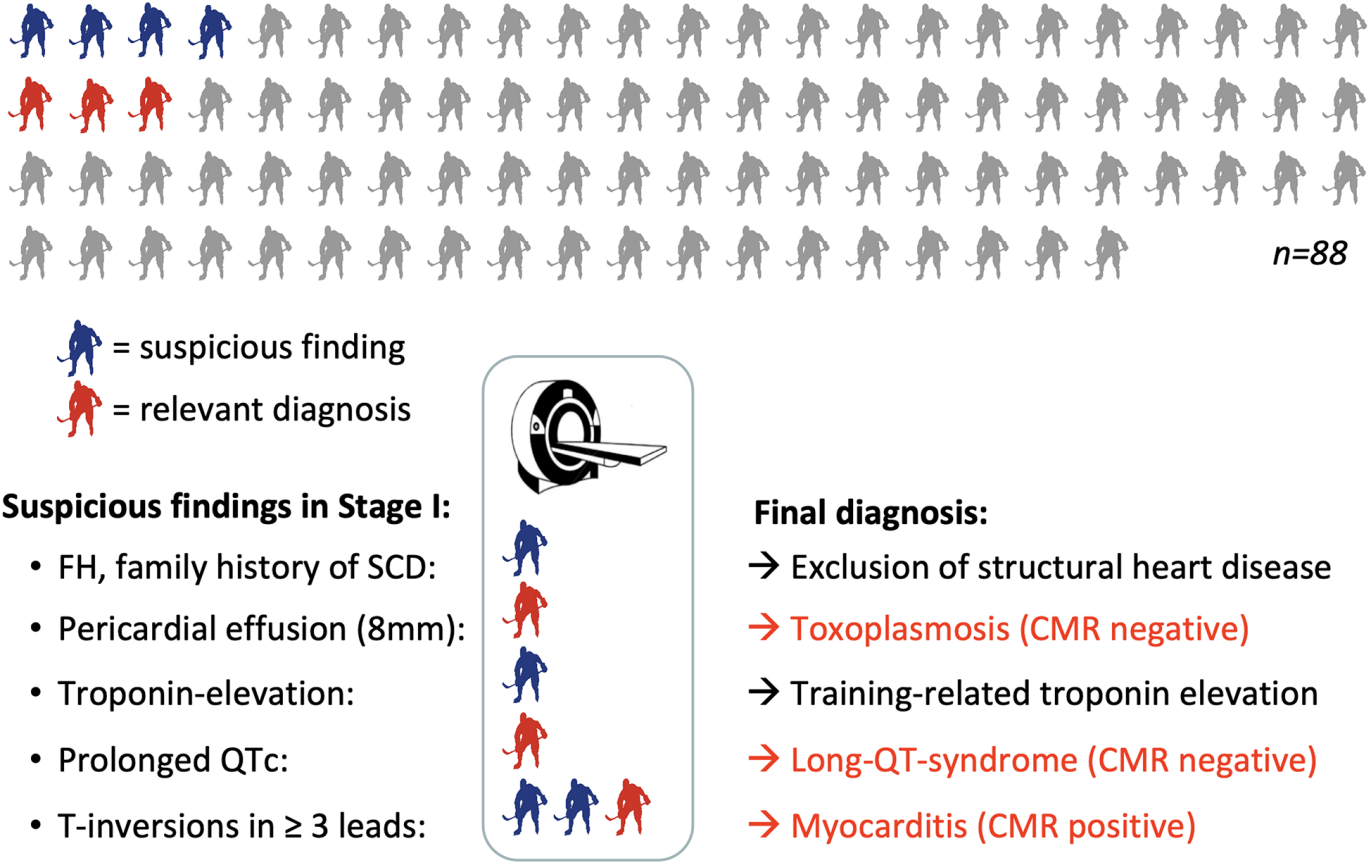
Graphical summary of the final results including cardiac MRI*.* Grey: player without pathologies at stage I; red: players with relevant diagnosis; blue = players with pathological findings in stage I showing normal results at stage II. CMR, cardiac magnetic resonance imaging; FH, family history; SCD, sudden cardiac death.

Eighty-eight elite female ice hockey players were enrolled between 01/2008 and 01/2018, at a median age of 16 (Table 2). The median time of follow-up from first screening to last contact was 13 years. Completeness of follow-up (FU) with at least ≥12 months individual follow-up was 100%. Main exercise related parameters are shown in Figure 2. All players included in this study were members of the German Female National Ice Hockey Teams.

**Table 2:** Baseline characteristics of athletes at stage I of the screening protocol.

	*N* = 88
Age (years)	16 ± 1; 13–23
Height (m)	1.66 ± 0.07; 1.54–1.80
Weight (kg)	62.5 ± 10; 47–87
BMI (kg/m^2^)	22.5 ± 2.1; 17.8–28.7
Competitive ice hockey (years)	10.0 ± 4; 2–14
Family history of SCD (%)	1 (1.1%)
Asthma bronchiale (%)	12 (13.6%)
Pathological physical examination (%)	7 (8%)
12-lead ECG	
Pathological 12-lead ECG (%)	4 (4.5%)
HR (/min.)	70 ± 16; 42–121
PQ interval (ms)	140 ± 20; 84–220
QT interval Bazett (ms)	420 ± 31; 320–513
Incomplete RBBB (%)	1 (1.1%)
Delta wave (%)	0 (0%)
Transthoracic echocardiography	
LVEF (%)	60 ± 0; 53–75
LVEDD (mm)	44.9 ± 3.4; 36–53
LA (mm)	30 ± 5; 24–36
IVS (mm)	8 ± 1; 7–11
sPAP (mmHg + CVP)	18 ± 8; 0–30
TAPSE (mm)	26 ± 3; 21–32
Wall motion abnormalities	0 (0%)
Pericardial effusion	1 (1.1%)
Holter Studies	
Mean HR, holter (/min)	80 ± 16; 60–118
Min. HR, holter (/min)	44 ± 6; 33–59
Max. HR, holter (/min)	158 ± 40; 92–206
Number of atrial premature contractions during holter	105 ± 235; 0–932
Number of ventricular premature contractions during holter	1 ± 7; 0–2,687
Athletes with >/= 1 ventricular couplet	2 (2.3%)
Athletes with >/= pauses >3 s	0 (0%)
Non-sustained ventricular tachykardia	0 (0)
Ventricular tachykardia	0 (0)
Laboratory tests	
Hemoglobin (g/L; 4.5–10.5)	13.8 ± 0.8; 10.7–15.8
Hämatokrit (%; 36.0–48.0)	41.4 ± 2.4; 34.4–46.9
C-reactive protein (mg/dl; <2.8)	0.4 ± 0.6; 0.06–10
Creatinine (mg/dl; <0.9)	0.8 ± 0.1; 0.7–1.1
Creatine Kinase (U/L; <123)	180 ± 20; 100–220
Significant troponin I value (%)	1 (1.1%)
LDL (mg/dl; <100)	92 ± 30; 34–182
Exercise tests	
Max. HR, ergometry (/min)	186 ± 10; 159–209
Max. systolic pressure, ergometry (mmHg)	200 ± 30; 278–150
Maximum workload capacity (watt)	190 ± 45; 275–160
Weight adjusted max. workload (watt/kg)	3.2 ± 1; 2–5
Aerobic threshold (watt)	97 ± 25; 60–160
Anaerobic threshold (watt)	142.2 ± 21.8; 95–210
Cardiac output at rest (L/min)	7.1 ± 1.5; 3.8–10.1
Cardiac output at 100 watt (L/min)	12.3 ± 1.7; 6.9–16.4
Max. cardiac output (L/min)	15.6 ± 3.5; 10.1–24.8
VO2max (ml/min/kg)	37.8 ± 5; 26.6–48.2
Maximal lactate (Mmol/L)	7.6 ± 3.2; 4.4–12.9

Table 2 gives the characteristics determined at baseline. Numbers are presented with percentages, the statistical mean is given with standard deviation (±SD), or median is given with interquartile range (±IQR); range as minimum and maximum is provided in addition.

BMI, body mass index; CVP, central venous pressure; ECG, electrocardiogram; HR, heart rate; IVS, interventricular septum; LA, left atrium; LDL, low density lipoprotein; LVEDD, left ventricular end diastolic diameter; LVEF, left ventricular ejection fraction; RBBB, right bundle branch block; SCD, sudden cardiac death; sPAP, systolic pulmonary artery pressure; TAPSE, tricuspid annular plane systolic excursion.

### Screening for SCD

In stage I of the screening model, seven of 88 athletes (8%) showed relevant findings (Figure 2). Following the study protocol, stage II of the screening model became applicable and one further non-invasive diagnostic approach was performed, which was a cardiac magnetic resonance (CMR) in all of the seven cases. Stage III of the study protocol (invasive diagnostics) was neither indicated nor performed in any of the participants.

**Figure 3:**
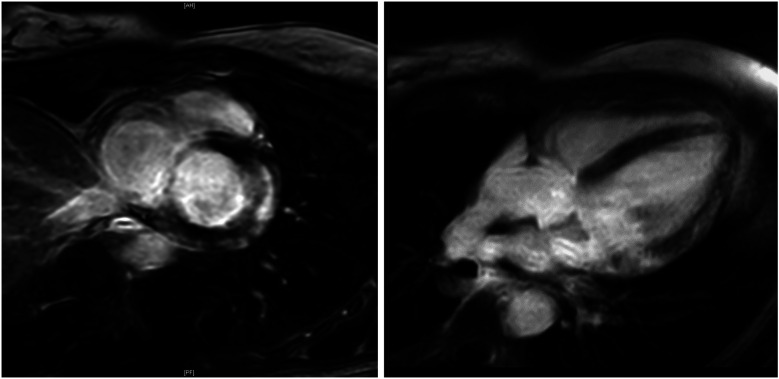
Cardiac MRI. Cardiac MRI of a young female ice-hockey player demonstrating late gadolinium enhancement of the lateral wall and global edema, following ECG-detected T-wave inversions, strongly suggesting myocarditis. MRI, magnetic resonance imaging.

**Figure 4:**
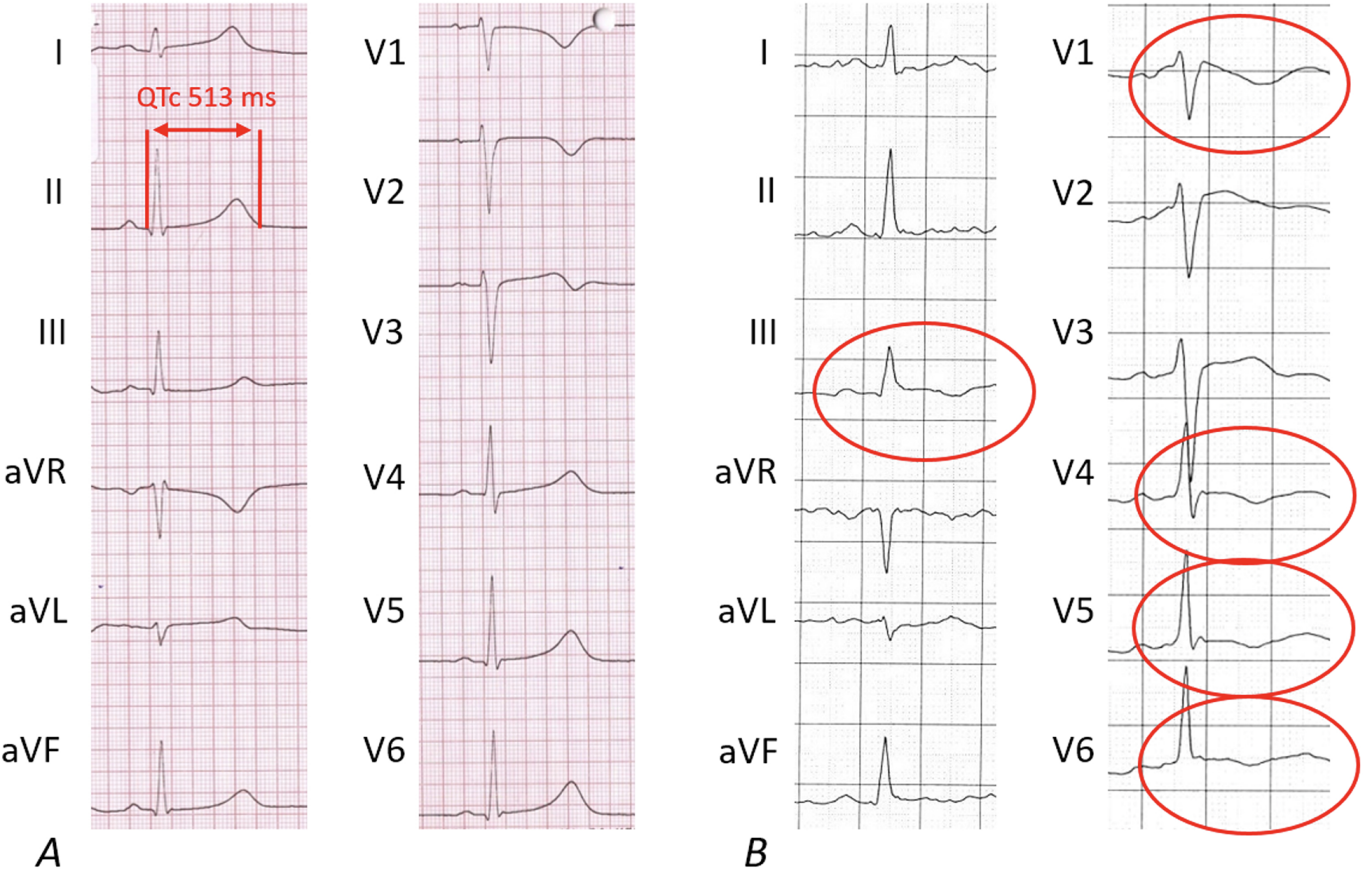
Pathological findings of 12-lead-electrocardigrams*.* The pathological 12-lead-ECGs from 2 out of 4 players lead to relevant diagnoses. **(A)** Shows the ECG of an athlete with a newly diagnosed Long-QT ECG pattern; **(B)** shows inverted T-waves in more than 2 leads leading to the final diagnosis of acute myocarditis following cardiac MRI.

At stage I, ECG alterations were the main relevant finding (4/7, 57%). Three of them had significant T-negativity in ≥3 ECG leads (Figure 3), so the cardiac MRI as part of stage II was applied and lead to the diagnosis of myocarditis in one athlete (Figure 3) while the other 2 did not show any pathologies. The player with confirmed myocarditis was advised to take a break from sports practice, treated with nonsteroidal anti-inflammatory drugs and angiotensin-converting-enzyme inhibitors and controlled after 4–6 weeks and 3 months. One out of four athletes with pathological ECG showed a significant prolonged QT interval (QT 536 ms, QTc Bazett 513 ms; Figure 4). In combination with a positive family history for prolonged QT intervals, a congenital Long-QT-syndrome was diagnosed and consequently an exclusion from competitive sports was recommended. The cardiac MRI (stage II) in this player showed no relevant findings.

There were no pathological electrocardiographic findings noted during spiroergometry.

In one athlete, the echocardiography (stage I) showed an asymptomatic pericardial effusion of a maximum of 8 mm. In this case, the cardiac MRI excluded myocarditis and revealed pericarditis. The potential cause of the pericarditis was found by serological examination: Positive IgM-antibody tests for toxoplasmosis with low IgG avidity were found. The player was treated with antibiotics, controlled with echocardiography several times and cleared for training and competition after full resolution of the pericardial effusion and seroconversion. One player with mildly elevated Troponin I, which was most likely related to excessive physical training, showed no pathologic findings in the following examinations and was cleared for training and competition. The family history showed familiar hyperlipidemia combined with history of SCD in one participant, who was also allowed to continue with competitive sports as all other screening examinations showed no relevant results.

In this study, the number needed to screen (NNTS) for the ECG to detect cardiovascular pathologies potentially leading to SCD was 44 with a sensitivity of 67% and specificity of 98%. Together with the echocardiography, the cumulative NNTS was 29. Two out of four participants (50%) showed false positive results at the 12-lead-ECG, but were allowed to continue with competitive sports as stage II excluded relevant pathologies.

The overall prevalence of conditions associated with an increased risk of SCD, which required a sport practice break or even an exclusion from competitive sports, was 3/88 players (3.4%). Four out of seven players (57%) with pathological findings in stage I showed no relevant finding in stage II and where allowed to continue with sports.

### Spiroergometry, Holter-ECG, echocardiography

**Figure 5:**
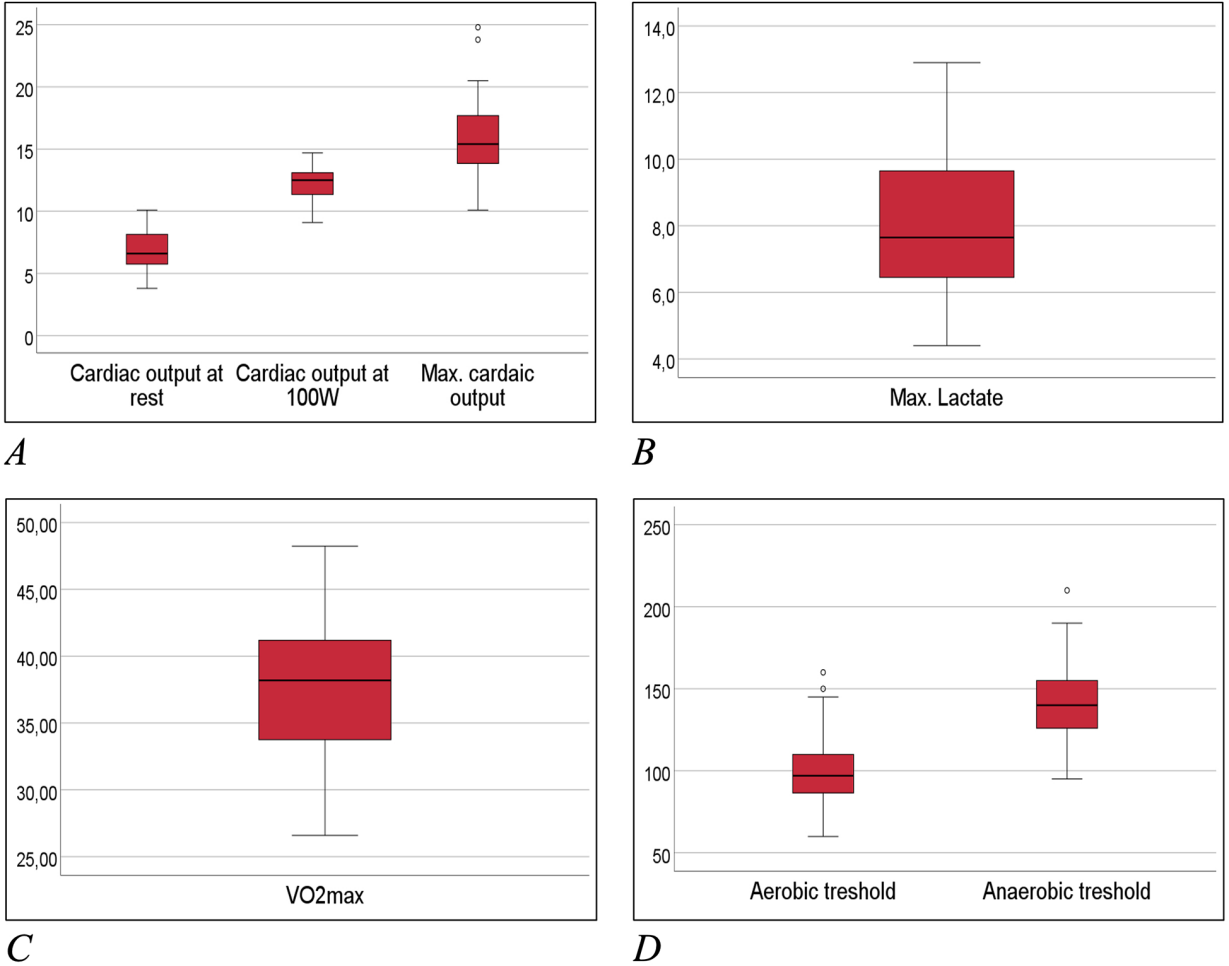
Box-whisker-plots of main exercise related measures. **(A)** Cardiac output at spiroergometry in ml/min; **(B)** lactate at maximum workload in mmol/L; **(C)** maximum oxygen uptake (Vo2max) in ml/min/kg; **(D)** aerobic and anaerobic threshold in watt.

Table 2 and Figure 5 depicts all important parameters of spiroergometry, lactate testing, Holter-ECG and echocardiography.

### Follow-up results

No case of SCD occurred during the total follow up of 13 years and 968 athlete years. One athlete (1/88, 1.1%) experienced acute pulmonary embolism during competition following long-distance traveling and was counted as MACCE, one athlete died from non-cardiac cause (cancer).

## Discussion

### Risk of SCD in female athletes

The preparticipation screening revealed a prevalence of 3.4% out of 88 participants showing 3 different pathologies that are associated with an increased risk of SCD. Considering the unique characteristics of our study population, which includes only young elite female ice hockey players, this rate appears to be relatively high. However, this finding aligns with results from young male athletes (3, 15, 16), demonstrating that preparticipation in sports to detect potential risks for sudden cardiac death is a highly relevant issue for women as well. This emphasizes the importance of conducting preparticipation tests regularly, regardless of gender.

In this population (peri-)myocarditis was the most common cardiac pathology followed by channelopathy (Long-QT), which is different from the most common causes of SCD in male athletes (2, 3). This gender difference in the etiology of sudden cardiac death in sports was also postulated in other studies, which suspected channelopathies as the most common causes in female athletes, while HCM predominates in men (2, 3, 7). Differences in hormonal mechanisms, sympathetic tone, catecholamine release, and the prevalence of hereditary diseases may result in varying causes and incidence rates of Sudden Cardiac Death (SCD) between male and female athletes (6, 8, 17).

In our study, mild and asymptomatic myocarditis emerged as the most prevalent pathological finding during screening. Considering that myocardial inflammation is relatively common in athletes and can lead to serious complications, including SCD, a comprehensive evaluation of the patient, including MRI and even endomyocardial biopsy, is crucial for effective management. This thorough assessment provides vital information for safely determining sports eligibility and estimating prognosis (18). For instance, a 2012 study by Gruen et al. indicated that 9.9% of patients with biopsy-confirmed myocarditis experienced SCD during a 5-year follow-up (19).

### 12-lead-ECG in preparticipation testing

This analysis emphasizes the crucial role of the 12-lead ECG in pre-participation testing to identify athletes at potential risk for Sudden Cardiac Death (SCD). With a reasonable number needed to screen (NNS 44), and high overall specificity of 98%, it substantiates its usage for detecting young female athletes who might be at risk for SCD.

Without the use of the ECG, neither the athlete with acute myocarditis nor the one with Long QT Syndrome would have been identified as “at risk” using only the other diagnostic tools available in Stage I.

Two out of four participants with pathological findings in the 12-lead EKG were able to continue participating in sports since no relevant findings were discovered during the additional examinations in stage I and II. This supports the idea that multi-stage screening models can aid in preventing false disqualifications.

Indeed, the effectiveness of the ECG largely depends on the expertise of the physician interpreting the results. Therefore, it is crucial to employ consistent ECG interpretation criteria during preparticipation screening (20, 21).

### Further examinations in preparticipation testing and in staged protocols

In addition to the 12-lead EKG, medical history, and clinical status, a transthoracic echocardiography is only recommended for patients with known structural heart diseases or as deemed necessary (22). However, the player with the pericardial effusion related to a toxoplasmosis would not have been detected without the use of the echocardiography as a “baseline” test at stage I during the screening. This supports a generous use of the echocardiography to improve the sensitivity of preparticipation screening models.

During Stage II, an MRI was performed on all athletes who exhibited any pathologies in Stage I. This resulted in MRIs being administered to 7 out of 88 athletes (13%), with one case confirming myocarditis. This rate appears relatively high, suggesting a need for stricter MRI indications within general pre-participation screening models. This would ensure applicability and effectiveness across all sport types.

A cardiac computed tomography (a stage II option) was not administered in this young population due to the absence of any indication.

According to the present recommendations, the ECG exercise test and a Holter-ECG can also be added to preparticipation screening as indicated (e.g., for athletes with symptoms or in elderly athletes >40) but as expected in the present young female subgroup, both did not contribute to the diagnosis of SCD related cardiac conditions.

### Reference data for female ice hockey athletes

Reference data for the baseline evaluation of female elite ice hockey players in preparticipation screening is rarely available. The present results helps to define standards in young female athletes and may be cited as reference values for healthy subject evaluation.

The spiroergometry by inert gas rebreathing (Innocor®) and the lactate tests as part of stage I of the protocol were not able to improve the sensitivity of screening for athletes “at risk for SCD” and could be omitted in further preparticipation screenings beyond academic studies. However, they can be useful for training control. To the best of our knowledge, this is the first report presenting VO2max, cardiac output under exercise and an aerobic and anaerobic threshold in the special group of young (median age 16 years) female ice hockey players. Certainly, there exists data on average reference values for endurance tests in elite athletes and hockey players, but these are primarily for male athletes over the age of 20 (23, 24). In the present study, VO2max was 37.8 ± 5 ml/min/kg in females elite ice hockey players at the mean age of 16 years. In comparison, in male ice hockey players at the age of 24, the VO2max was higher (51.8 ± 3 ml/min/kg) (24). Diameters and dynamic measurements from echocardiography as part of stage I match to normal reference values in adults (25, 26) but do not exceed them like as consequence of training-related cardiovascular adaptations (e.g., enlargement of LV dimensions, wall thickness) seen in male ice hockey players and athletes of other sports (27, 28). This might be explained by the very young population and the cardiac impact of testosterone in male athletes (8).

Therefore, the existing dataset could be used for comparison in future analyses of similar subgroups, and may also serve as a clinically relevant reference for the evaluation of young female patients.

### Limitations

The main limitation of the study is the small sample size and the short follow-up time, especially because SCD in young female athlete is known to have a low incidence rate. As the analyzed population was quite specific including only young female ice hockey players (median age 16), the results might not be generalizable to other sports, age, or gender. No comparative group exists with non-screened athletes. Fortunately, no SCD occurred in the follow-up of this study.

Thus, we are unable to link our findings directly to the critical endpoint of mortality. We can only hypothesize that the screening model detected precursory irregularities related to sudden cardiac death (SCD), especially since no SCD occurred during the follow-up period of the study.

## Conclusions

This study highlights the need for comprehensive preparticipation screening, especially 12-lead ECG, in young female elite ice hockey players to detect potential risk for sudden cardiac death (SCD). In this group, there is a significant 3.4% prevalence of conditions which could lead to SCD. Both ECG and echocardiography proved effective in detecting cardiac conditions like congenital Long QT syndrome and myocarditis. Subsequent cardiac MRI application also enabled accurate evaluations. No SCD cases occurred during follow-up, demonstrating the screening's effectiveness. The study underlines the need for ongoing vigilance and tailored screenings considering female athletes' unique SCD pathophysiological profiles and etiologies. The findings advocate for including comprehensive cardiac screening in young female athletes' preparticipation evaluations, enhancing their safety across all sports disciplines.

## Data Availability

The datasets presented in this article are not readily available due to the sensitive nature of the clinical data involved in this study, and in accordance with the ethical standards and institutional guidelines. This decision is made to ensure the privacy and confidentiality of patient information, as well as to comply with the institutional policies on data protection and patient consent. Researchers interested in accessing the raw data for verification purposes or for conducting further analyses are encouraged to contact the corresponding author. Requests to access the datasets should be directed to florian.straube@muenchen-klinik.de.
